# Diffuse Pleural Mesothelioma Initially Presenting as Cervical Lymphadenopathy: A Report of a Rare Case

**DOI:** 10.7759/cureus.114875

**Published:** 2026-08-20

**Authors:** Kaushik Saha, Sayantika Ghosh, Soumita Poddar

**Affiliations:** 1 Pathology, M. L. Jain Diagnocare, Berhampore, IND; 2 Pathology, Murshidabad Medical College and Hospital, Berhampore, IND; 3 Radiotherapy, Murshidabad Medical College and Hospital, Berhampore, IND

**Keywords:** fine-needle aspiration cytology (fnac), immunohistochemistry, lymph node, malignant mesothelioma, metastatic

## Abstract

Diffuse mesothelioma is a rare malignant tumor arising from mesothelial cells, showing diffuse involvement of the pleura and having a strong association with asbestos exposure. Cervical lymphadenopathy as the initial presentation of pleural mesothelioma is very unusual to date. We report here such a rare case of diffuse pleural mesothelioma in a 42-year-old male patient who primarily presented with left-sided lower cervical lymphadenopathy. Fine-needle aspiration cytology (FNAC) from the lymph nodes demonstrated hypercellular smears with polygonal epithelioid cells arranged mostly as singly scattered cells, loosely cohesive clusters, monolayered pavement-like sheets, and spheroid groups. Many cells displayed binucleation, multilobed nuclei, cytoplasmic vacuoles, fuzzy cell borders, and mitotic figures. Guided tru-cut biopsy from the lymph node revealed diffuse sheets of atypical epithelioid cells with abundant eosinophilic cytoplasm, with large nuclei and prominent nucleoli in places. The tumor cells were immunopositive for pancytokeratin, CK5/6, EMA, Calretinin, D2-40, and WT1 while negative for all other relevant markers. Diffuse, irregular thickening and nodularity of left-sided pleura and mediastinal lymphadenopathy were identified on contrast-enhanced computed tomography (CECT). It is very important to consider metastatic pleural mesothelioma in the differential diagnosis of lymph node mass on FNAC, especially in the cervical region, to avoid misdiagnosis or delay in the correct diagnosis of this strikingly unusual presentation.

## Introduction

Pleural mesotheliomas are rare and aggressive cancers that account for <2% of all malignancies. The majority of mesotheliomas are caused by occupational exposure to asbestos. It is much more common in elderly men, but it has also been reported in younger age groups [1]. A wide range of benign and malignant lesions of the pleura need to be excluded to reach the final diagnosis due to overlapping morphologic and immunophenotypic features [1,2]. Mesothelioma was commonly encountered and discussed in effusion cytology specimens [1,3]. However, very few cases have been presented with initial cervical lymphadenopathy, as lymph node involvement other than mediastinal lymph nodes is very uncommon [1]. We report here such a rare case of diffuse pleural mesothelioma in a 42-year-old man who primarily presented with cervical lymphadenopathy, where lymph node cytology provided a valuable clue for further diagnostic workup for mesothelioma.

## Case presentation

**Figure 1:**
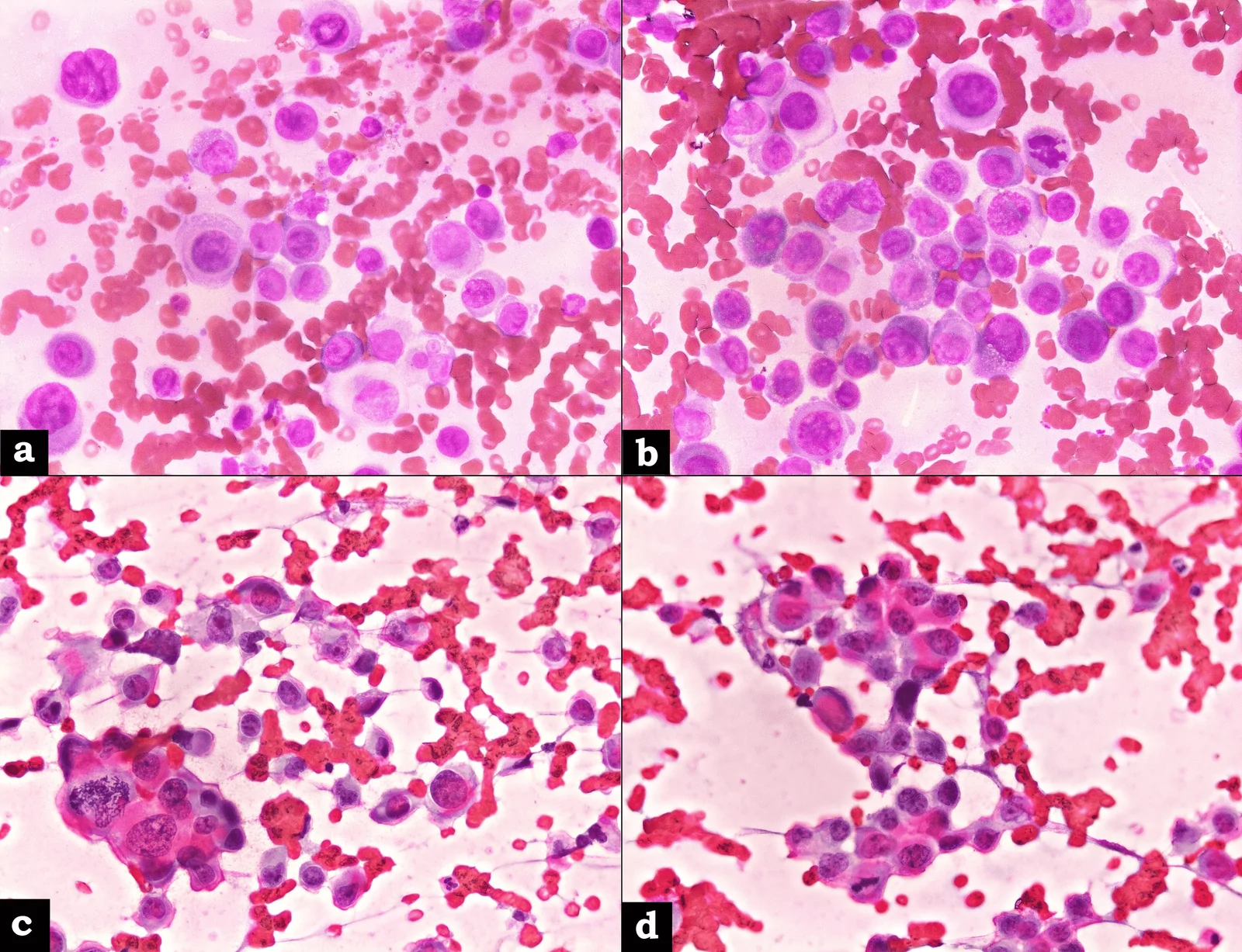
Photomicrographs ((a) May-Grünwald Giemsa (MGG), ×400; (b) MGG, ×400; (c) Pap, ×400; and (d) Pap, ×400) showing numerous polygonal epithelioid cells as singly scattered cells, loosely cohesive clusters, and monolayered pavement-like sheets of cells with a low N:C ratio, abundant cytoplasm, central to eccentric nuclei, prominent nucleoli, binucleation, multilobed nuclei, cytoplasmic vacuoles, fuzzy cell border, and mitotic figures.

A 42-year-old male construction worker presented with left-sided lower cervical lymphadenopathy. He had no other complaints such as respiratory distress or chest pain. The consultant oncologist immediately referred the patient for fine-needle aspiration cytology (FNAC) to rule out a malignant lymphoproliferative lesion. Cytology (Figure 1) revealed hypercellular smears with numerous polygonal epithelioid cells arranged mostly as singly scattered cells, loosely cohesive clusters, monolayered pavement-like sheets, and spheroid groups.

**Figure 2:**
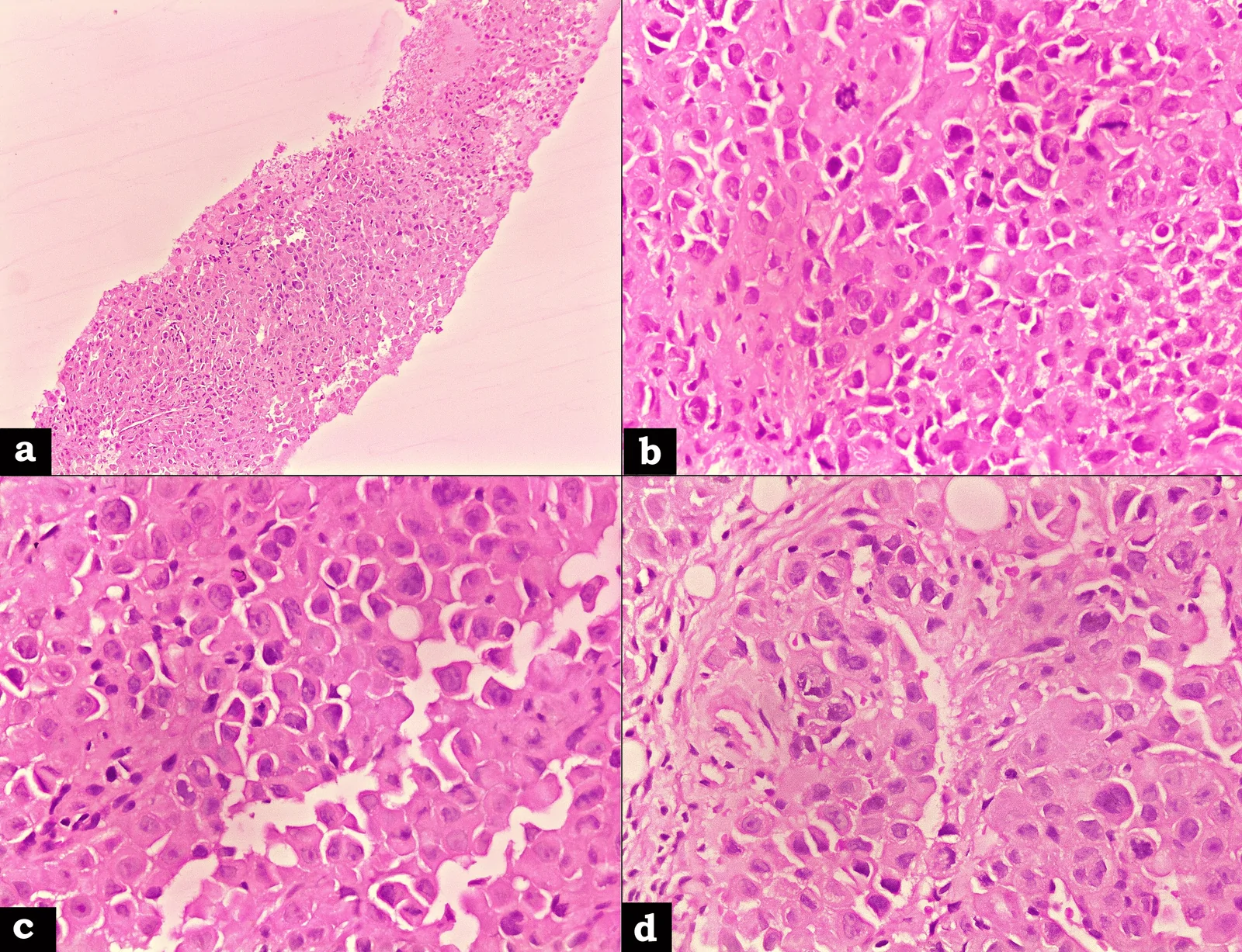
Photomicrograph showing (a) low-power view (H&E, ×100) and (b-d) high-power view (H&E, ×400) of sheets of atypical epithelioid cells with large nuclei, anisonucleosis, and prominent nucleoli. Focal acinar pattern, intracytoplasmic vacuoles, and large histiocytoid discohesive cells are also seen.

Cells had well-defined cell borders, low nuclear-to-cytoplasmic ratio, moderate to abundant dense cytoplasm, central to eccentric round to oval nuclei with finely granular chromatin, prominent central nucleoli, and moderate anisonucleosis. Many cells displayed binucleation, multilobed nuclei, cytoplasmic vacuoles, fuzzy cell borders, and mitotic figures. Overall cytological features favored an epithelioid malignant tumor, and the possibility of lymphoid malignancies was almost excluded. Ultrasound sonography (USG)-guided tru-cut biopsy (Figure 2) from the lymph node demonstrated diffuse sheets of atypical epithelioid cells with abundant eosinophilic cytoplasm, having large nuclei, anisonucleosis, scattered tumor giant cells, and prominent nucleoli in places.

**Figure 3:**
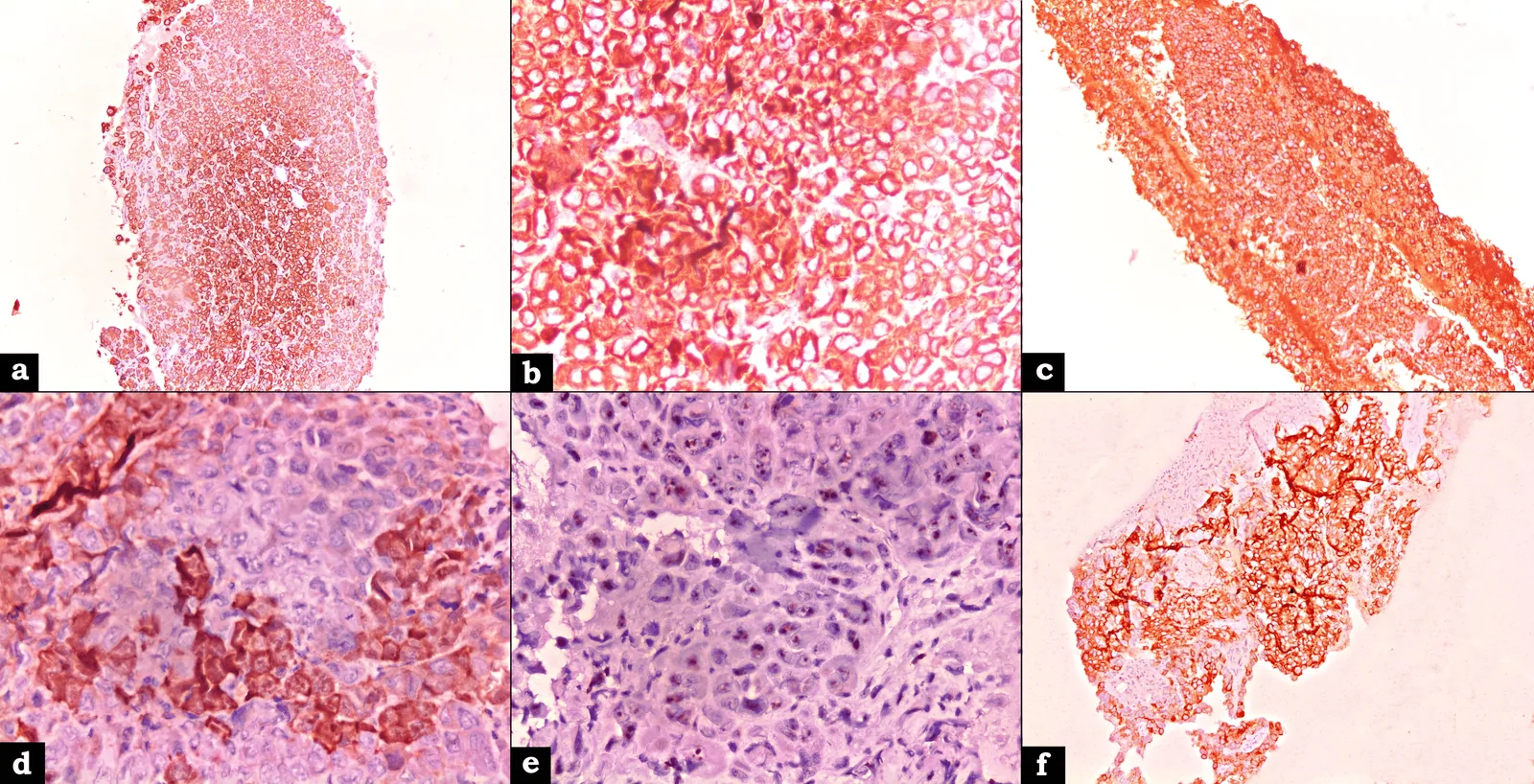
Positive immunostaining of malignant cells with (a) pancytokeratin (×100), (b) CK5/6 (×400), (c) EMA (×100), (d) Calretinin (×400), (e) WT1 (×400), and (f) D2-40 (×100).

Focal acinar pattern, intracytoplasmic vacuoles, microcysts, and large histiocytoid discohesive cells were noted. The tumor cells were immunopositive (Figure 3) for pancytokeratin, CK5/6, EMA, Calretinin, WT1, and D2-40 while negative for all other relevant markers like CD45, CD20, TdT, CD3, CD23, Vimentin, CD34, PAX8, BCL2, TLE1, SOX10, CD68, GATA3, CDX2, TTF1, p40, CD56, S100, and Synaptophysin to exclude the differential diagnoses.

Diffuse, irregular thickening and nodularity of left-sided pleura with mild ipsilateral pleural effusion and mediastinal lymphadenopathy were identified on contrast-enhanced computed tomography (CECT). Positron emission tomography with 18F-fluorodeoxyglucose (FDG PET/CT) showed high FDG accumulation throughout the pleural lesion and in the lymph nodes.

## Discussion

Diffuse mesothelioma (DM) is a tumor of malignant mesothelial cells showing diffuse involvement of the pleura or pericardium and with poor long-term survival. Morphologically, it may be epithelioid, sarcomatoid including desmoplastic, or biphasic type [1,2]. Initial presenting features of DM usually include dyspnea, unilateral chest pain or pleural effusion, cough, or constitutional symptoms [1,2]. Malignant mesothelioma presenting with peripheral lymphadenopathy as the initial symptom is exceptionally rare [4]. Very few cases have been reported where metastatic lymphadenopathy was the initial clinical presentation [4,5]. Priya et al. [6] and Tafazzoli et al. [7] cytologically detected metastasis in supraclavicular lymph nodes in a symptomatic patient with pleural mesothelioma, while Kant et al. [8] demonstrated a case of cervical lymph node metastasis with pleuritic chest pain. Ogata et al. [9] diagnosed a case with recurrent pleural effusion from cervical lymph node biopsy. Zhang et al. [4] presented three cases, and Sussman and Rosai [10] reported six cases of systemic lymphadenopathy as initial presentation of mesothelioma. Craig et al. [11] diagnosed a case of axillary lymph node metastasis from occult pericardial mesothelioma by FNAC.

Epithelioid mesothelioma, as in our case, should be distinguished from metastatic carcinomas including clear cell carcinoma of the kidney, melanoma, and epithelioid sarcomas. Similarly, lymphoma and other small round cell tumors in cases of small cell predominant lesions, all types of spindle cell lesions including sarcomatoid carcinoma in cases of sarcomatoid type, and sometimes many benign lesions of pleura are considered in the differential diagnosis [1,2]. No single histopathological feature or immunohistochemical (IHC) marker is enough to diagnose mesothelioma confidently due to its overlapping histological and IHC features with many other pleural lesions. That is why a panel of IHC markers with clinic-radiological correlation is necessary to establish the diagnosis [1,2].

## Conclusions

It is challenging for a pathologist to consider the diagnosis of malignant mesothelioma when it exceptionally presents only with extrathoracic lymph node metastasis prior to the discovery of the primary tumor in the pleura. Whenever a pathologist confronts a metastatic epithelioid neoplasm in a lymph node from an unknown primary site, malignant mesothelioma should be kept in the list of potential differential diagnoses. Cytology from the lymph node can provide a clue in the prompt diagnosis with further investigations.
